# 

**Published:** 1858-04

**Authors:** 


### SANITARY REVIEW,

AND

### Journal of Public Health.

EDITED BY

BENJAMIN W. RICHARDSON, M.D.,

LICENTIATE OF THE ROYAL COLLEGE OF PHYSICIANS,
PHYSICIAN TO THE ROYAL INFIRMARY FOR DISEASES OF THE CHEST, AND LECTURER
ON PUBLIC HYGIENE AT THE GROSVENOR PLACE MEDICAL SCHOOL.

NATIONAL HEALTH IS NATIONAL WEALTH.

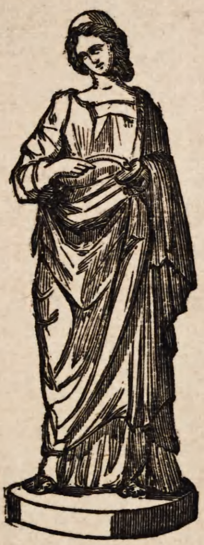

'ΥΓΙΕΙΑ

*πρεσβίστη μακάρων.*

VOL. IV.

PRINTED AND PUBLISHED FOR THE PROPRIETOR BY

THOMAS RICHARDS, 37, GREAT QUEEN STREET.

1858.

WI
SA672P

### BOOKS RECEIVED.

Annual Report of the British Meteorological Society. London : 1858.

Barnes (R., M.D.) Annual Report of the Medical Officer of Health for Shoreditch, for the year ending 26th December, 1857. London : 1858.

Bigg (H. H.) On the Mechanical Appliances necessary for the Treatment of Deformities. Part I. The Lower Limbs. London : Churchill. 1858.

Brown-Sequard (M. E.) Sur les Lois de l'Irritabilité Musculaire, de la Rigidité Cadavérique, et de la Putréfaction (Extrait des Comptes rendus de l'Académie des Sciences.) Paris : 1857.

Brown-Sequard (M. E.) Recherches expérimentales sur les Propriétés physiologiques du Sang chargé d'oxygène et du Sang chargé de l'acide carbonique. (Extrait des Comptes rendus de l'Académie des Sciences.) Paris : 1858.

Cox (W. I.) The Diseases of Colliery Operatives. London : Richards : 1857.

Gamgee (John.) On the Study of Veterinary Medicine, being an Inaugural Address at the opening of the Edinburgh new Veterinary School. Edinburgh : Sutherland and Knox. 1857.

Glaisher (James, F.R.S.) On the Meteorology of England during the Quarter ending December 31, 1857. London : 1857.

Glaisher (James, F.R.S.) On the Meteorology of Scotland during the Quarter ending December 31, 1857.

Griffith (J. W., M.D.) General Report on the Sanitary State of Clerkenwell for 1856. London : 1857.

Hitchman (John.) The Virtues of Salt as a Remedy for the Cold or Collapsed Stage of Cholera. Extract of a Letter to the *Leamington Spa Courier.*

Hunt (Thomas.) A Guide to the Treatment of Diseases of the Skin, for the use of the Student and General Practitioner. Illustrated by Cases. London : Churchill. 1857.

Inman (T., M.D.) The Phenomena of Spinal Irritation, and other Functional Diseases of the Nervous System, explained, and a Rational Plan of Treatment deduced. London : Churchill. 1858.

Inman (T., M.D.) The Nature of Inflammation, and the Principles on which it should be treated, examined from a common-sense point of view. To which is added, a History of Atheroma in Arteries : its Nature and Alliances. Liverpool : H. Greenwood.

Jones (T. Wharton, F.R.S.) A Catechism of the Physiology and Philosophy of Body, Sense, and Mind. For use in Schools and Colleges, and in Private Study. London : Churchill. 1858.

Knox (R., M.D., F.R.S.E.) Man : his Structure and Physiology ; popularly Explained and Demonstrated. With eight moveable dissected coloured plates, and five woodcuts. London : Baillière. 1857.

Lankester (E., M.D.) Directions for the Preservation of Health and the Prevention of the Spread of Catching or Infectious Diseases. Westminster : 1858.

Lardner (Dionysius, D.C.L.) Animal Physiology for Schools. With 190 Illustrations. London : Walton and Maberly. 1858.

429514

Mackay (G., M.D.) Notes on the Cholera at Varna in 1854, and more especially in Her Majesty's Ship *Agamemnon,* in the Black Sea, between the 1st August, 1854, and 8th September, 1855. Edinburgh : Murray and Gibb. 1857.

Marcet (W., M.D.) On the Immediate Principles of Human Excrements in the Healthy State. (From the *Philosophical Transactions.*) London : 1857.

Miller (James, F.R.S.E.) Alcohol : its Place and Power. Glasgow : *Scottish Temperance League.* 1858.

Milton (J. L.) On Scirrhus of the Male Breast. (From the *Medico-Chirurgical Transactions.*) London : 1858.

Mortality Tables for the Parish of Croydon, for the years ending December 31, from 1848 to 1857, inclusive.

Murphy (Edward W., M.D.) What is Puerperal Fever? A Question proposed to the Epidemiological Society of London. Dublin : M'Glashan and Gill. 1857.

Nourse (W. E. C.) A Short and Plain History of Cholera ; its Causes and Prevention ; written for Popular Use. London : Churchill. 1858.

Paine (H. J.) Fifth Annual Report on the Sanitary Condition of Cardiff. Cardiff : 1858.

Rumsey (Henry W.) On Sanitary Legislation and Administration in England. London : Churchill. 1858.

Sieveking (E. H., M.D.) On Epilepsy and Epileptiform Seizures : their Causes, Pathology, and Treatment. London : Churchill. 1858.

Thomson (R. D., M.D., F.R.S.) Annual Report on the Health of the Parish of St. Marylebone, year 1856. London : 1857.

Yearsley (James.) Controversy on the Artificial Tympanum ; Reprinted from the *Medical Times and Gazette,* with Additions. London : H. Baillière, 1858.

---

### IN EXCHANGE.

Literary Gazette.
Psychological Journal.
British Medical Journal.
Ranking and Radcliffe's Abstract of the Medical Sciences.
Chemist.
Veterinarian.
Asylum Journal of Mental Science.
Quarterly Journal of the Chemical Society.
Scottish Review.
Edinburgh Medical Journal.
Glasgow Medical Journal.
Dublin Hospital Gazette.
American Journal of the Medical Sciences.
New York Journal of Medicine.
Montreal Medical Chronicle.
Charleston Medical Journal.
Gazette Médicale de Paris.
Aerzliches Intelligenz-Blatt.
Medical and Surgical Reporter.
Journal of Practical Medicine and Surgery.
Journal de Physiologie.

### NOTES AND CORRESPONDENCE.

The Editor is anxious to give free scope to the expression of opinion, but will not hold himself responsible for the opinions of those of his contributors who attach their names to the papers.

Dr. Brown-Sequard's Journal de Physiologie. The appearance of a Journal of Physiology under the editorial supervision of so distinguished a physiologist as Dr. Brown-Sêquard, is, in the medical world, the literary event of the quarter. The learned editor of this new work, seeing the bearings of physiology on the great facts of preventive medicine, admits into his numbers papers in which the connection of the two sciences is practically recognized. We heartily recommend this valuable periodical to all readers, professional and general, who wish to keep on a level with the rapid advances of physiological science.

The Fothergillian Prize. Our readers will be gratified to hear that the Fothergillian Gold Medal has this year been awarded by the Council of the Medical Society of London to our esteemed friend and contributor Dr. Herbert Barker, of Bedford, for his Essay on "The Influence of Malaria and Miasmata in the Production of the following Diseases: Typhus and Typhoid Fevers, Cholera, and the Exanthemata." We give in this number of the Sanitary Review a paper from Dr. Barker's pen, in which a brief sketch is supplied of the subject matter of this Essay.

Testimonial to Dr. McWilliam. We have great pleasure in observing that a testimonial from his professional brethren has been presented to Dr. McWilliam, the Honorary Secretary of the Epidemiological Society, in recognition of his immense labours in the naval medical service.

Epidemic Preaching Mania in Sweden. The interesting letters on the Epidemic Preaching Mania in Sweden which appeared in the *Morning Post* under the signature F.R.S., and of which an extract appeared in the *British Medical Journal* for January 16th, were written by Dr. John Webster, whose papers on various sanitary subjects have often appeared in the pages of this Review. We have been prevented by want of space from referring at length to these letters in the present number.

---

### To New Subscribers to the Sanitary Review.

The Publisher begs to intimate, that new Subscribers wishing to have the series of the Journal complete from the first number, can obtain sets of Vols. I, II, and III, at Subscribers' prices, 10s. each, post free, by application to him, enclosing Post-office Order, payable at the Charing Cross Branch to Thomas Richards.

The Sanitary Review and Journal of Public Health is sold by all Booksellers, and is equally suited for the general and professional reader, for the scientific society, and for the private library.

London: T. Richards, 37, Great Queen Street.

### TRANSACTIONS

OF THE

### EPIDEMIOLOGICAL SOCIETY

### OF LONDON

FOR THE YEAR 1858.

LONDON:
T. RICHARDS, 37, GREAT QUEEN STREET.

M.DCCC.LVIII.

*Is now Published, price* 16*s.*

### THE ASTLEY COOPER PRIZE ESSAY,

ON THE

### CAUSE OF THE

### COAGULATION OF THE BLOOD:

WITH A PRACTICAL APPENDIX.

BY

BENJAMIN W. RICHARDSON, M.D., L.R.C.P.,

PHYSICIAN TO THE ROYAL INFIRMARY FOR DISEASES OF THE CHEST.

---

**Contents:**

CHAPTER I. Historical Memoranda on the supposed Causes of Coagulation of the Blood.

CHAPTER II.—Cause of the Phenomenon of Coagulation: present position of the Question.

CHAPTER III.—The Author's Personal Observations and Deductions on the Condition of the Blood in the Body under certain Physiological and Pathological States.

CHAPTER IV.—Experimental Inquiry into the Physical Agencies influencing the Coagulation of the Blood.

CHAPTER V.—Experimental Inquiry into the Chemical Agencies influencing the Coagulation of the Blood.

CHAPTER VI.—Experimental Inquiry into the Chemical Agencies influencing the Coagulation of the Blood (continued).

CHAPTER VII.—Phenomenon of the Buffy Coat.

CHAPTER VIII.—Summary of Conclusions and Propositions.

APPENDIX.

1.—Superalkaline Conditions of the Blood in Relation to Disease.

2.—Ammonia as an Exhalation from the Body.

3.—Experiments, shewing the Effect of Lactic Acid on Animal Bodies (with Coloured Plates, shewing the stages of Endocarditis).

4.—Deposition of Fibrin in the Heart and Blood-vessels during Life.

5.—The Alkalies and Acids as Remedial Agents.

6.—Application of Ammonia to the Experiment of Transfusion of Blood.

7.—On some Conditions of the Blood after Death, in relation to Medico-Legal Inquiries.

8.—List of Authors.

*The Essay is Illustrated with numerous Wood Engravings, and with Coloured Plates by Tuffen West.*

London: JOHN CHURCHILL, New Burlington Street.

